# Maternal and Neonatal Outcomes Associated With Preeclampsia and Gestational Diabetes Mellitus: A Retrospective Cohort Study

**DOI:** 10.7759/cureus.86120

**Published:** 2025-06-16

**Authors:** Abdullah Umer, Saadia Kanwal, Angbeen Ahmad, Arooj Fatima, Adeeba Fatima, Fatima Farooq, Noor Fatima, Sumbal Jawad

**Affiliations:** 1 Radiology, Combined Military Hospital, Lahore, Lahore, PAK; 2 Obstetrics and Gynecology, Shalamar Hospital, Lahore, PAK; 3 Obstetrics and Gynecology, Services Institute of Medical Sciences, Lahore, PAK; 4 Obstetrics and Gynecology, Lady Willingdon Hospital, Lahore, PAK; 5 Obstetrics and Gynecology, Divisional Headquarters (DHQ) Teaching Hospital, Mirpur, Mirpur, PAK; 6 Internal Medicine, Fauji Foundation Hospital, Lahore, Lahore, PAK; 7 Obstetrics and Gynecology, Divisional Headquarters (DHQ) Teaching Hospital, Gujranwala, Gujranwala, PAK

**Keywords:** gestational diabetes mellitus, maternal outcomes, neonatal outcomes, preeclampsia, pregnancy

## Abstract

Background

Preeclampsia and gestational diabetes mellitus (GDM) are common pregnancy complications associated with significant maternal and neonatal morbidity.

Objectives

This study aims to evaluate and compare maternal and neonatal outcomes in pregnancies complicated by preeclampsia, GDM, and the coexistence of both conditions.

Methods

This retrospective observational study included 230 pregnant women who delivered at Shalamar Hospital, Lahore, Pakistan, between January 2023 and December 2024. The participants were divided into three groups: preeclampsia only (n = 80), GDM only (n = 75), and both conditions (n = 75).

Results

The coexistence of preeclampsia and GDM was associated with the highest rates of cesarean section (82.7%, n = 62), preterm birth (46.7%, n = 35), neonatal intensive care unit (NICU) admission (49.3%, n = 37), and neonatal hypoglycemia (18.7%, n = 14). Maternal complications, including intensive care unit (ICU) admission (10.7%, n = 8) and postpartum hemorrhage (9.3%, n = 7), were also more frequent in this group. Logistic regression confirmed that combined preeclampsia and GDM independently predicted adverse outcomes such as cesarean delivery (odds ratio {OR}: 2.4), NICU admission (OR: 2.1), and preterm birth (OR: 1.9).

Conclusion

It is concluded that the co-occurrence of preeclampsia and GDM significantly worsens both maternal and neonatal outcomes compared to either condition alone. Early diagnosis, multidisciplinary management, and targeted interventions are crucial to mitigating these risks and improving perinatal care.

## Introduction

Preeclampsia and gestational diabetes mellitus (GDM) are among the most common and critical complications that arise during pregnancy, both contributing significantly to adverse maternal and neonatal outcomes globally. The various medical challenges of each disorder share certain aspects because they commonly connect through risk element analysis and pathophysiological processes and extended effects [1]. The ongoing worldwide increase in metabolic disorders, together with cardiovascular diseases, has made it necessary to better understand pregnancy-related conditions and their potential health outcomes [2]. Preeclampsia functions as a pregnancy-specific multi-amenable hypertensive condition that develops after week 20 of gestation [3]. Medical experts define preeclampsia as when blood pressure elevations occur with proteinuria, together with organ dysfunction signs from hepatic, renal, and neurological aspects [4]. Acute hypertensive disorder manifests during pregnancy in 2%-8% of women across the globe, thus becoming a major contributor to maternal deaths among low-income nations worldwide. The condition may advance toward fatal complications such as eclampsia; hemolysis, elevated liver enzymes, and low platelet count (HELLP) syndrome; placental abruption; and posterior reversible encephalopathy syndrome (PRES) when healthcare management is not provided [5]. Among these, posterior reversible encephalopathy syndrome (PRES) is a rare but serious neurological condition characterized by seizures, altered consciousness, and visual disturbances, typically identified via neuroimaging. PRES has been increasingly recognized as a severe manifestation of uncontrolled or rapidly progressing preeclampsia, particularly in the postpartum period [6]. Neonates born to preeclampsia mothers face increased dangers of intrauterine growth restriction (IUGR), preterm birth, and stillbirth, and these conditions result in persistent negative impacts on neonatal to childhood development [7].

The prevalence of gestational diabetes mellitus (GDM) in pregnant women ranges from 5% to 20% according to separate population research bases and diagnostic parameter methods. The condition develops when insulin resistance and pancreatic β-cell dysfunction exist together, although these natural pregnancy changes become overly pronounced in susceptible people [8]. GDM produces various maternal risks that lead to the raised potential of cesarean delivery, as well as preeclampsia, alongside type 2 diabetes later in mothers. The health issues for newborns become severe as GDM raises their odds of facing fetal macrosomia, alongside shoulder dystocia, birth trauma, respiratory distress syndrome, and neonatal hypoglycemia [9]. Metabolic syndrome and obesity, alongside type 2 diabetes, develop in both mother and child during their lifespan. Recent research suggests that GDM and preeclampsia co-occurring during pregnancy produce an additional level of risk that worsens complications affecting mothers and their babies [10]. A woman with GDM has elevated risks for developing preeclampsia when glycemic control remains insufficient alongside obesity or existing chronic hypertension [11]. Maternal preeclampsia exists together with GDM, which makes glycemic management and pregnancy monitoring more demanding because intensive maternal care usually triggers premature delivery [12]. The pathophysiological links between these conditions include impaired endothelial health, together with persistent inflammation and oxidative stress and hormone system disruptions that cause damage to the placenta. Vascular health deterioration through these mechanisms limits proper placental blood circulation and reduces supplies of nutrients and oxygen to the fetus [13]. The co-prevalence of preeclampsia and gestational diabetes mellitus (GDM) is rising globally, yet evidence on their combined impact on maternal and neonatal health remains limited. Clarifying the additive risks of these comorbid conditions is critical to inform targeted perinatal monitoring, risk stratification, and clinical management.

This study aims to evaluate and compare maternal and neonatal outcomes in pregnancies complicated by preeclampsia, GDM, and the coexistence of both conditions.

## Materials and methods

Study design and setting

This retrospective observational study was conducted at Shalamar Hospital, Lahore, Pakistan. Patients were enrolled from January 2023 to December 2024. A total of 230 pregnant women were included in the study.

**Figure 1:**
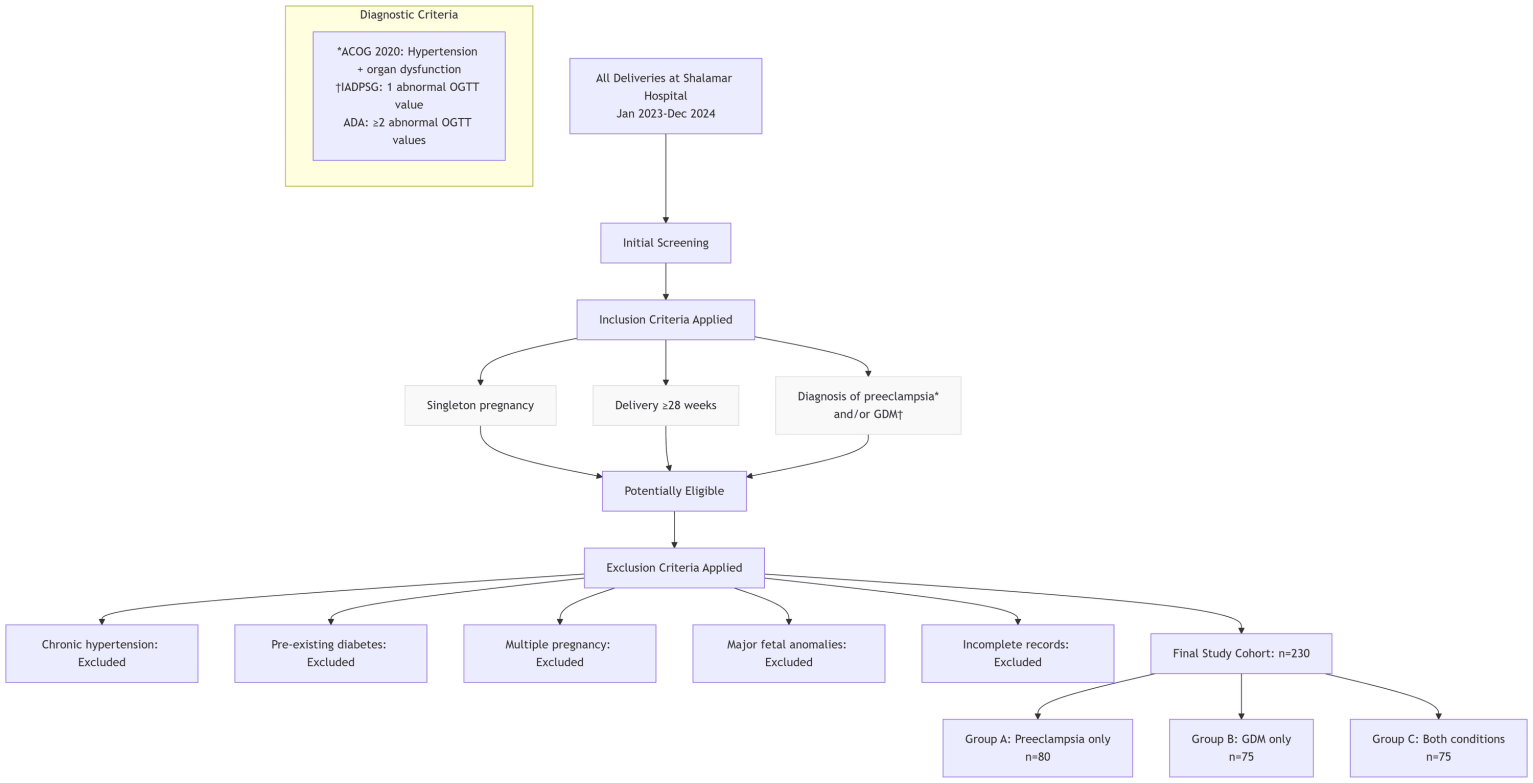
Study Flowchart: Cohort Selection Process *Preeclampsia diagnosis was based on ACOG 2020 criteria †GDM diagnosis was based on either IADPSG criteria or ADA criteria IADPSG, International Association of Diabetes and Pregnancy Study Groups; ACOG, American College of Obstetricians and Gynecologists; OGTT, oral glucose tolerance test; GDM, gestational diabetes mellitus; ADA, American Diabetes Association

The participants were selected based on medical records and classified into three groups: Group A, patients diagnosed with preeclampsia only (n = 80); Group B, patients diagnosed with GDM only (n = 75); and Group C, patients with both preeclampsia and GDM (n = 75) (Figure 1).

Inclusion and exclusion criteria

**Figure 2:**
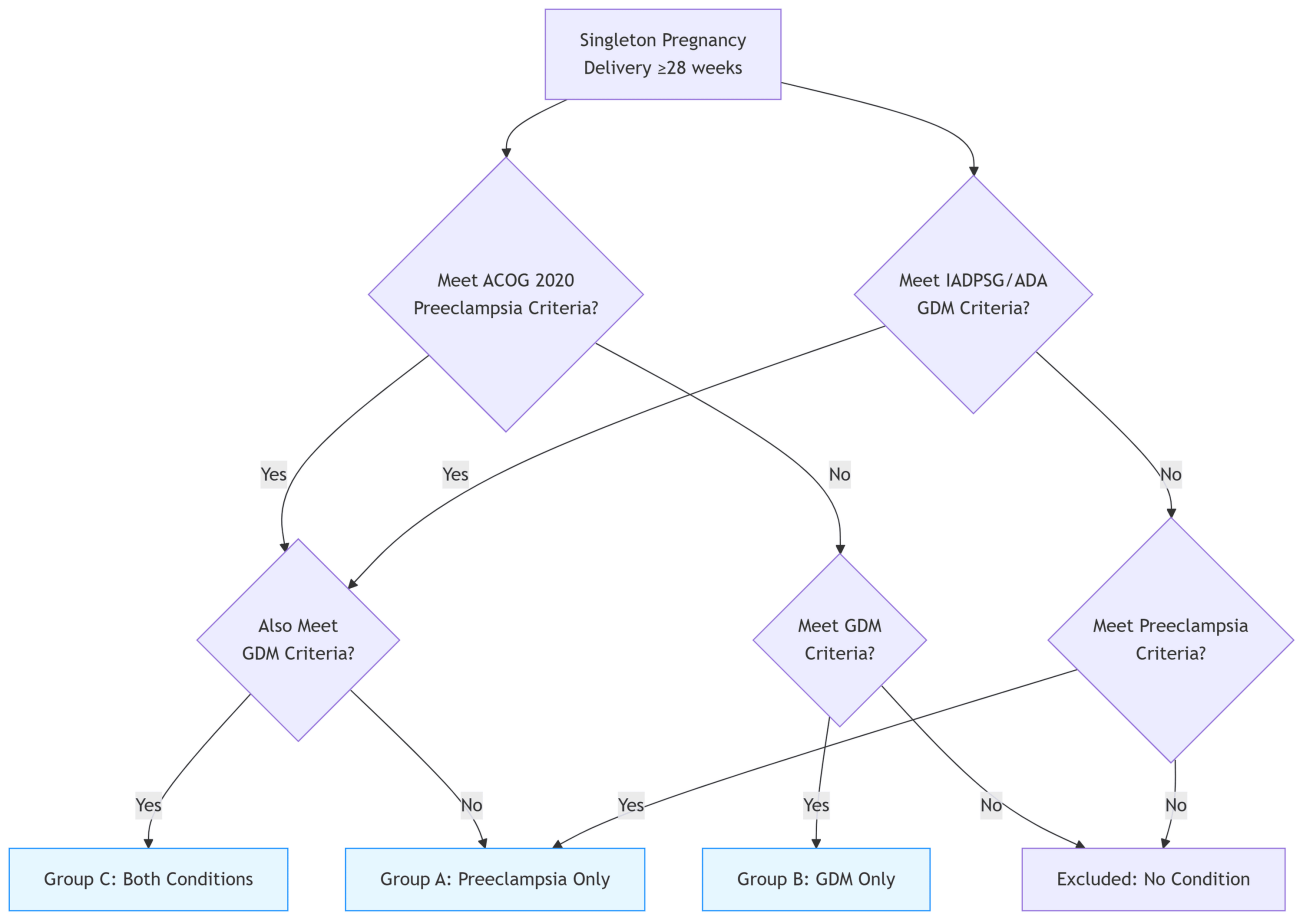
Diagnostic Classification Algorithm IADPSG, International Association of Diabetes and Pregnancy Study Groups; ACOG, American College of Obstetricians and Gynecologists; GDM, gestational diabetes mellitus; ADA, American Diabetes Association

Eligible participants were those with a singleton pregnancy diagnosed with preeclampsia according to the American College of Obstetricians and Gynecologists (ACOG) 2020 criteria [14] and gestational diabetes mellitus (GDM) based on either the International Association of Diabetes and Pregnancy Study Groups (IADPSG) [15] or American Diabetes Association (ADA) [16] criteria, who delivered at 28 weeks of gestation or later. Individuals were excluded if they had preexisting chronic hypertension or type 1 or type 2 diabetes, a multiple pregnancy, major fetal anomalies, or incomplete medical records (Figure 2).

Data collection

Patient data were obtained retrospectively from the hospital's electronic medical records and supplemented by manual chart reviews where necessary. A standardized data abstraction form was used to ensure uniformity in data extraction. Maternal data included demographic characteristics such as age, gravidity, parity, and body mass index (BMI), as well as clinical variables such as gestational age at diagnosis and delivery, blood pressure and blood glucose levels, antenatal complications, mode of delivery, and the use of medications such as insulin or antihypertensives. Maternal complications including eclampsia, HELLP syndrome, postpartum hemorrhage, and intensive care unit (ICU) admission were also documented. Neonatal data included birth weight, Apgar scores at one and five minutes, gestational age at delivery, admission to the neonatal intensive care unit (NICU), and neonatal complications such as hypoglycemia, macrosomia, respiratory distress syndrome, and perinatal mortality.

Statistical analysis

Data analysis was performed using IBM SPSS Statistics for Windows, version 26.0 (IBM Corp., Armonk, NY; released 2019). Descriptive statistics were utilized to summarize patient characteristics. Categorical variables are presented as frequencies (N) and percentages (%), while continuous variables are shown as means ± standard deviation (SD) or medians with interquartile ranges (IQR), as appropriate. Comparative analyses among the three study groups were conducted using the chi-square test for categorical variables and one-way ANOVA or t-test for continuous normally distributed variables. A p-value of less than 0.05 was considered statistically significant.

## Results

Data were collected from 230 patients. The mean maternal age was 30.7 ± 5.1 years in the preeclampsia with GDM group, 30.1 ± 4.5 years in the GDM-only group, and 29.8 ± 4.7 years in the preeclampsia-only group. Body mass index was highest in women with both conditions (31.8 ± 4.1), followed by the GDM group (30.4 ± 3.7) and the preeclampsia group (29.1 ± 3.2). Gravidity and parity were also greater in the combined group (median: 3 {IQR: 2-3} and 2 {IQR: 1-3}, respectively) compared to the other two groups. The proportion of primigravida women was 55.0% (n = 44) in the preeclampsia group, 48.0% (n = 36) in the GDM group, and 42.7% (n = 32) in the combined group. A family history of diabetes was reported by 36.0% (n = 27) in the combined group, 30.7% (n = 23) in the GDM group, and 18.8% (n = 15) in the preeclampsia group. Previous hypertension was noted in 41.3% (n = 33) of the preeclampsia group, 38.7% (n = 29) of the combined group, and 22.7% (n = 17) of the GDM group (Table 1).

**Table 1:** Demographic and Baseline Characteristics Data are represented as mean ± SD, median (IQR), or n (%) BMI, body mass index; GDM, gestational diabetes mellitus; SD, standard deviation; IQR, interquartile range

Demographic Variable	Preeclampsia Only (n = 80)	GDM Only (n = 75)	Preeclampsia + GDM (n = 75)
Mean Maternal Age (Years)	29.8 ± 4.7	30.1 ± 4.5	30.7 ± 5.1
BMI (Mean ± SD)	29.1 ± 3.2	30.4 ± 3.7	31.8 ± 4.1
Gravidity (Median, IQR)	2 (1-3)	2 (1-2)	3 (2-3)
Parity (Median, IQR)	1 (0-2)	1 (0-2)	2 (1-3)
Primigravida (n, %)	44 (55.0%)	36 (48.0%)	32 (42.7%)
Family History of Diabetes (n, %)	15 (18.8%)	23 (30.7%)	27 (36.0%)
History of Hypertension (n, %)	33 (41.3%)	17 (22.7%)	29 (38.7%)

The mean maternal age was similar across groups, ranging from 29.8 to 30.7 years (p = 0.09). A higher proportion of women with both preeclampsia and GDM had a BM of ≥30 (62.7%, n = 47) compared to those with GDM only (49.3%, n = 37) and preeclampsia only (45.0%, n = 36), showing statistical significance (p = 0.04). Gestational age at delivery was lowest in the combined group (36.2 ± 1.8 weeks) and highest in the GDM-only group (37.3 ± 1.4 weeks), with a significant difference (p = 0.01). Cesarean delivery was most frequent among women with both conditions (82.7%, n = 62), compared to 68.7% (n = 55) in the preeclampsia group and 60.0% (n = 45) in the GDM group (p = 0.02). Eclampsia occurred in 8.0% (n = 6) of the combined group and 3.7% (n = 3) of the preeclampsia group but was absent in the GDM group. HELLP syndrome was observed only in the group with both conditions (6.7%, n = 5) (Table 2).

**Table 2:** Maternal Outcomes Data shown as mean ± SD or n (%). ANOVA was used for continuous variables and the chi-square test for categorical variables. Significance level: p < 0.05 GDM, gestational diabetes mellitus; ICU, intensive care unit; BMI, body mass index; SD, standard deviation; HELLP, hemolysis, elevated liver enzymes, and low platelet count

Maternal Outcome	Preeclampsia (n = 80)	GDM (n = 75)	Preeclampsia + GDM (n = 75)	Test Statistic	P-value
Mean Maternal Age (Years)	29.8 ± 4.7	30.1 ± 4.5	30.7 ± 5.1	F = 2.45	0.09
BMI ≥ 30 (n, %)	36 (45.0%)	37 (49.3%)	47 (62.7%)	χ² = 6.47	0.04
Gestational Age at Delivery (Weeks)	36.9 ± 1.6	37.3 ± 1.4	36.2 ± 1.8	F = 4.95	0.01
Cesarean Section (n, %)	55 (68.7%)	45 (60.0%)	62 (82.7%)	χ² = 7.65	0.02
Eclampsia (n, %)	3 (3.7%)	0 (0.0%)	6 (8.0%)	-	-
HELLP Syndrome (n, %)	0 (0.0%)	0 (0.0%)	5 (6.7%)	-	-
ICU Admission (n, %)	4 (5.0%)	0 (0.0%)	8 (10.7%)	-	-
Postpartum Hemorrhage (n, %)	4 (5.0%)	2 (2.7%)	7 (9.3%)	χ² = 6.95	0.03

The incidence of preterm birth was highest in the preeclampsia with GDM group (46.7%, n = 35), followed by preeclampsia only (35.0%, n = 28) and GDM only (21.3%, n = 16), with the difference being statistically significant (p < 0.001). Low birth weight was most frequently observed in the combined group (38.7%, n = 29) and least in the GDM group (17.3%, n = 13). NICU admission rates were significantly higher among neonates born to mothers with both conditions (49.3%, n = 37), compared to 35.0% (n = 28) and 26.7% (n = 20) in the preeclampsia and GDM groups, respectively (p = 0.004). Neonatal hypoglycemia occurred most commonly in the preeclampsia with GDM group (18.7%, n = 14) and in GDM alone (14.7%, n = 11), with minimal incidence in the preeclampsia group (2.5%, n = 2). Macrosomia was most prevalent in the GDM group (13.3%, n = 10), followed by the combined group (9.3%, n = 7) and preeclampsia group (2.5%, n = 2) (p = 0.01). Intrauterine growth restriction (IUGR) was more frequent in the preeclampsia group (15.0%, n = 12) and the combined group (12.0%, n = 9) while least common in the GDM group (3.3%, n = 3). Perinatal mortality was highest among neonates in the combined group (5.3%, n = 4), followed by preeclampsia only (3.7%, n = 3) and GDM only (1.3%, n = 1), although this difference did not reach statistical significance (p = 0.08) (Table 3).

**Table 3:** Neonatal Outcomes Values expressed as n (%). The chi-square test was used for all comparisons. Significance threshold: p < 0.05 GDM, gestational diabetes mellitus; IUGR, intrauterine growth retardation; NICU, neonatal intensive care unit

Neonatal Outcome	Preeclampsia (n = 80)	GDM (n = 75)	Preeclampsia + GDM (n = 75)	Test Statistic	P-value
Preterm Birth (n, %)	28 (35.0%)	16 (21.3%)	35 (46.7%)	χ² = 15.27	<0.001
Low Birth Weight (n, %)	24 (30.0%)	13 (17.3%)	29 (38.7%)	-	-
NICU Admission (n, %)	28 (35.0%)	20 (26.7%)	37 (49.3%)	χ² = 11.03	0.004
Neonatal Hypoglycemia (n, %)	2 (2.5%)	11 (14.7%)	14 (18.7%)	-	-
Macrosomia (n, %)	2 (2.5%)	10 (13.3%)	7 (9.3%)	χ² = 8.96	0.01
IUGR (n, %)	12 (15.0%)	3 (3.3%)	9 (12.0%)	-	-
Perinatal Mortality (n, %)	3 (3.7%)	1 (1.3%)	4 (5.3%)	χ² = 4.89	0.08

Maternal age of over 35 years was observed in 32.0% (n = 24) of women with both preeclampsia and GDM, compared to 26.3% (n = 21) in the preeclampsia-only group and 25.3% (n = 19) in the GDM group, though this was not statistically significant (p = 0.18). A family history of diabetes was significantly more common in the combined group (36.0%, n = 27) and GDM group (30.7%, n = 23) than in the preeclampsia group (18.8%, n = 15) (p = 0.02). Similarly, previous GDM was reported in 34.7% (n = 26) of the combined group and 28.0% (n = 21) of the GDM group but absent in those with preeclampsia alone (p < 0.001). The prevalence of prior hypertension was higher in the preeclampsia group (41.3%, n = 33) and the combined group (38.7%, n = 29) compared to the GDM group (22.7%, n = 17) (p = 0.04) (Table 4).

**Table 4:** Risk Factor Distribution All variables presented as n (%). The chi-square test was used for group comparisons. A p-value of <0.05 indicates statistical significance GDM, gestational diabetes mellitus; BMI, body mass index

Risk Factor	Preeclampsia (n = 80)	GDM (n = 75)	Preeclampsia + GDM (n = 75)	Test Statistic	P-value
Maternal Age > 35 (n, %)	21 (26.3%)	19 (25.3%)	24 (32.0%)	χ² = 3.49	0.18
Family History of Diabetes (n, %)	15 (18.8%)	23 (30.7%)	27 (36.0%)	χ² = 7.81	0.02
History of Hypertension (n, %)	33 (41.3%)	17 (22.7%)	29 (38.7%)	χ² = 6.43	0.04
Previous GDM (n, %)	0 (0.0%)	21 (28.0%)	26 (34.7%)	χ² = 26.94	<0.001
Obesity (BMI ≥ 30) (n, %)	36 (45.0%)	37 (49.3%)	47 (62.7%)	χ² = 6.41	0.04

The odds of cesarean section were significantly higher in this group, with an adjusted odds ratio (OR) of 2.4 (95% confidence interval {CI}: 1.3-4.6; p = 0.004). Similarly, the likelihood of neonatal intensive care unit (NICU) admission was elevated (OR: 2.1; 95% CI: 1.1-3.9; p = 0.02). Preterm birth was also significantly associated with the combined condition, with an OR of 1.9 (95% CI: 1.1-3.4; p = 0.03) (Table 5).

**Table 5:** Logistic Regression Summary Multivariate logistic regression results adjusting for maternal age, BMI, parity, and comorbidities. Odds ratios (ORs) with 95% confidence intervals (CI) are reported. Significance level set at p < 0.05 NICU, neonatal intensive care unit; BMI, body mass index

Outcome Variable	Adjusted OR	95% CI	P-value
Cesarean Section	2.4	1.3-4.6	0.004
NICU Admission	2.1	1.1-3.9	0.02
Preterm Birth	1.9	1.1-3.4	0.03

## Discussion

This study aimed to evaluate and compare the maternal and neonatal outcomes in pregnancies complicated by preeclampsia, gestational diabetes mellitus (GDM), and the coexistence of both conditions. Research results show that the combined occurrence of preeclampsia and GDM leads to intensified dangers for mothers and newborns above individual risks of each condition separately. Studies show that when preeclampsia and GDM exist together, women need more cesarean sections at rates reaching 82.7%, whereas single cases of preeclampsia and GDM yield lower C-section rates at 68.7% and 60%, respectively. The higher rate possibly stems from both clinicians' increased caution in medical care and from additional obstetric issues, which affect fetal distress and glycemic control and hypertensive crises [17]. Studies have found that maternal patients with combined vascular and metabolic conditions face increased risks for cesarean delivery due to a higher incidence of macrosomia, together with preterm labor and poor uterine blood flow [18].

Postpartum hemorrhage, along with eclampsia and HELLP syndrome, proved more prevalent among patients with dual diagnoses, thus increasing maternal morbidity rates based on this study's findings. Increased ICU admissions among these particular patients highlight the necessity for enhanced observation practices and quick response procedures [19]. Healthcare providers should note that infants born to mothers with GDM and preeclampsia experience premature delivery at rates of 46.7% and require NICU care in 49.3% of cases. Neonates born to mothers with both GDM and preeclampsia suffered increased occurrences of low birth weight and neonatal hypoglycemia. The research shows that placental insufficiency due to preeclampsia, together with metabolic conditions from GDM, results in combined negative effects on fetal development and newborn welfare. Studies show that the fetal growth pattern becomes unpredictable in women with both GDM and preeclampsia because the prevalence of macrosomia rises alongside increased cases of intrauterine growth restriction (IUGR) [20].

The research data indicate that co-occurring preeclampsia and GDM lead to a 2.4-fold increased risk for cesarean section, along with a 2.1-fold higher NICU admission probability and double risk for preterm delivery (OR: 2.1 and 1.9, respectively) when controlled for age and BMI and other demographic variables. Several other reports confirm that pregnancy complications caused by combined metabolic and vascular damage consistently lead to negative perinatal outcomes [21]. The evaluation of pregnancy risk elements demonstrated increased rates of maternal obesity along with older maternal age and diabetic history among female patients diagnosed with both preeclampsia and GDM [22]. The early detection of maternal risks and multidisciplinary care approaches must receive emphasis for high-risk pregnant women. The study outcomes spotlight strategies for disease prevention, together with crucial considerations about health systems. The disease burden reduction may benefit from preconception counseling, as well as weight management and early screening for both glucose intolerance and hypertension [23,24]. While pharmacological interventions play a role in managing gestational diabetes mellitus, non-pharmacological approaches remain the first-line strategy, especially in cases with elevated fasting glucose. Lifestyle modifications, including medical nutrition therapy and structured physical activity, have been shown to significantly improve glycemic control and pregnancy outcomes. These findings support the importance of comprehensive prenatal counseling and non-pharmacological interventions in managing GDM, especially in resource-limited settings [25]. The study results demonstrate the value in building antenatal care programs that unite metabolic and hypertensive disorder assessments through standardized screening and management plans.

While this study offers valuable insights into the comparative outcomes of preeclampsia, GDM, and their coexistence, several limitations must be acknowledged. The retrospective design introduces inherent risks of selection bias and information bias due to reliance on existing medical records, which may vary in completeness and accuracy. Although we excluded cases with significant missing data, we recognize that a more detailed assessment and the reporting of missing variables could have enhanced transparency. Additionally, the absence of a formal sample size calculation or power analysis limits our ability to definitively conclude on rare outcomes such as perinatal mortality or severe neonatal morbidity. The study's single-center setting may also affect the generalizability of our findings to other populations with different demographic or healthcare characteristics. Despite these limitations, the use of well-established diagnostic criteria and adjustment for key confounders through multivariate analysis strengthens the internal validity of our findings and underscores the need for future prospective, multicenter studies to build upon this evidence.

## Conclusions

It is concluded that the coexistence of preeclampsia and gestational diabetes mellitus (GDM) during pregnancy significantly increases the risk of adverse maternal and neonatal outcomes compared to either condition alone. Women with both conditions were more likely to undergo cesarean delivery, experience severe maternal complications such as postpartum hemorrhage and ICU admission, and deliver preterm. Neonates born to these mothers faced higher rates of NICU admission, low birth weight, and hypoglycemia.
